# Lifestyle perceptions following multimodal prehabilitation in patients undergoing cancer surgery: a qualitative study

**DOI:** 10.1007/s00520-026-10661-7

**Published:** 2026-04-21

**Authors:** J. M. van Koeveringe, L. van Afferen, L. D. Drager, S. Hermsen, F. Atsma, B. van den Heuvel, J. P. H. Seeger

**Affiliations:** 1 Department of Surgery, Radboud University Medical Center, Nijmegen, The Netherlands; 2 HAN University of Applied Sciences, Nijmegen, The Netherlands; 3 Prevention Hub, Radboud University Medical Centre, Nijmegen, The Netherlands; 4 Department of Health Sciences, IQ Healthcare, Radboud University Medical Center, Nijmegen, The Netherlands

**Keywords:** Lifestyle behavior, Multimodal prehabilitation, Perioperative period, Qualitative study, Abdominal cancer surgery, Behavioral support

## Abstract

**Background:**

Lifestyle behaviors play a pivotal role across all stages of cancer. Multimodal prehabilitation, incorporating exercise, nutrition, and psychological support before treatment, has demonstrated improvements for short-term postoperative outcomes. Yet, its potential to catalyze sustainable lifestyle change after surgery remains unexplored. Understanding how patients perceive lifestyle changes after the perioperative phase is key to fully realizing prehabilitation benefits and leveraging the teachable moment at diagnosis.

**Objective:**

To gain a deeper understanding of patients’ perceptions of lifestyle after undergoing surgery following a multimodal prehabilitation program.

**Methods:**

This phenomenological qualitative study was performed in a university hospital between December 2022 and September 2023. Sixteen patients who have undergone abdominal cancer surgery following Fit4Surgery participation were interviewed using semi-structured interviews and thematic analysis. All interviews were audio-recorded, transcribed verbatim, and analyzed using reflexive thematic analysis.

**Results:**

The analysis identified five key themes that illustrate how patients with abdominal cancer perceive lifestyle following major surgery: (1) multimodal prehabilitation as a catalyst for lifestyle change, (2) challenges in maintaining a healthy lifestyle post-surgery, (3) the role of motivation and support in sustaining lifestyle change, (4) meaning of health and well-being, and (5) unequal access and personal context influence lifestyle choices.

**Conclusion:**

Multimodal prehabilitation provides a foundation for lifestyle change, but without ongoing support and realistic timelines, sustained healthy behavior adoption remains challenging, especially for less active patients. Future programs should move beyond clinical endpoints to prioritize long-term behavior change by leveraging motivation, external support, habit formation, and addressing barriers like fatigue and insufficient medical guidance.

**Supplementary Information:**

The online version contains supplementary material available at 10.1007/s00520-026-10661-7.

## Introduction

There is growing recognition of the significant role lifestyle factors play across the entire cancer continuum, from disease onset and treatment response to rehabilitation, recurrence, survival, and overall well-being [1, 2]. A healthy lifestyle can enhance physical functioning, reduce treatment-related side effects, and improve psychosocial outcomes [3, 4]. Accordingly, healthcare professionals increasingly encourage lifestyle modifications among cancer patients and survivors to optimize clinical and personal outcomes [5, 6].

A cancer diagnosis is a profound life event that typically triggers emotional and cognitive shifts, making patients more receptive to adopting health-promoting behaviors [7]. This period of heightened awareness and willingness to change is known as a *teachable moment*, a critical window of opportunity to initiate and support behavior change. This moment presents an opportunity for interventions to be particularly effective in motivating patients to engage in healthier lifestyles [8].

Multimodal prehabilitation is an emerging strategy in the preoperative phase of cancer treatment that leverages this teachable moment. Prehabilitation aims to enhance patients’ physical and psychological readiness for surgery by targeting modifiable risk factors prior to the procedure. These programs typically involve intensive physical exercise, nutritional counselling, mental health support, and support for smoking cessation.

While the benefits of prehabilitation in improving short-term postoperative outcomes are increasingly documented, its potential to promote long-term lifestyle change after surgery remains underexplored. Gaining an in-depth understanding of how patients interpret and integrate healthy behaviors into their lives after the perioperative period is essential for designing interventions that extend beyond the hospital setting.

Given the potential of prehabilitation to enhance utilization of the “teachable moment” created by a cancer diagnosis towards sustainable lifestyle change, this qualitative study aims to gain a deeper understanding of patients’ perceptions of lifestyle after undergoing surgery following a multimodal prehabilitation program. Additionally, it aims to identify the factors that, from the patients’ perspective, facilitate or hinder their ability to sustain a healthy lifestyle after surgery.

## Methods

### Design

We conducted a qualitative study with a phenomenological orientation, using semi-structured interviews and reflexive thematic analysis (RTA) with patients who had undergone abdominal cancer surgery following Fit4Surgery (F4S) participation. The study was conducted in accordance with the Consolidated Criteria for Reporting Qualitative Research (COREQ) guidelines [9]. Ethical approval was obtained as part of the F4S PREHAB trial from the local Medical Ethics Committee (METC Oost-Nederland; NL73777.091.20).

### Researcher characteristics and reflexivity

LA (male, MSc), a physiotherapist with a master’s degree in musculoskeletal rehabilitation, conducted all interviews and performed the initial thematic analysis. He works in primary care where pre- and rehabilitation are part of clinical practice. His professional background may have influenced his focus on physical functioning and the value of prehabilitation. Coding and interpretation were further informed by LD (male, MD, MSc), providing a medical perspective on the surgical trajectory. Reanalysis was done by JvK (female, MSc), a public health scientist, who was pursuing a PhD focused on integrating prevention within clinical care pathways. LD and JvK both received qualitative training. JS (male, PhD), a senior researcher, participated in multiple sessions to deepen data engagement and challenge interpretations. Additionally, JS took an active role during the thematization phase to help construct and refine the final themes. The involvement of multiple coders enhanced the reflection and discussion from different perspectives to deepen understanding.

The researchers approached the study with the assumption that prehabilitation may positively influence lifestyle domains while long-term health behavior change remains challenging. None of the researchers had prior treatment relationships with participants. Reflexivity was maintained through iterative discussions and field notes (LA) taken after each interview to ensure transparency and context-aware interpretation.

### Sampling of participants

Patients aged 18 and older who underwent abdominal cancer surgery after completing the F4S program at Radboudumc were recruited using convenience sampling. To ensure diverse representation, stratification was applied across cancer types, including liver, pancreas, colon, rectum, and oropharynx. Recruitment occurred during two timeframes (Nov 2022–Jan 2023 and Jul–Sep 2023), with participants contacted chronologically and approached at least three months post-surgery. Exclusion criteria included palliative care or inability to understand Dutch.

Members of the research team contacted potential participants by phone. Written informed consent including permission to use pseudonymized data and quotations for publication was obtained prior to the interviews.

Consistent with the principles of RTA, sample size was based on data richness and conceptual depth, rather than data saturation. Interviews were reviewed iteratively in sets of four, with the research team assessing the depth and relevance of emerging meanings after each cycle to determine whether further recruitment was needed.

### Data collection and analysis

During the first period, ten semi-structured interviews were conducted in December 2022 and January 2023. In the second period between August 2023 and September 2023, six additional interviews were conducted to enrich the dataset continuing until the research team determined that the data possessed sufficient conceptual depth to address the research questions.

The interview guide included seven open-ended questions, with sub questions, informed by literature on physical and psychosocial behavior determinants affecting lifestyle adaptation [8]. The guide was discussed within the research team and piloted with two participants prior to final data collection. Initially, the interviews focused on the physical activity component of multimodal prehabilitation, but the researchers iteratively expanded the interview guide to include other lifestyle factors addressed in the program, such as nutrition, mental health, and substance use (see Electronic Supplementary Material).

All patients were interviewed for 30 to 45 min in a quiet and comfortable setting. At the end of each session, participants had the opportunity to reflect on the discussion and provide additional insights. The interviews were audio-recorded and transcribed verbatim using the Word package from Microsoft Office 365. Participants received their transcripts for review and were given the opportunity to provide comments or corrections.

Data were coded using Braun and Clarke’s [10] six-phase RTA approach to guide our analysis: data familiarization, coding, searching for themes, reviewing themes, defining and naming themes and writing-up the analysis. Three researchers independently coded the transcripts using ATLAS.ti (version 24.0), following an inductive approach. For this study, we consciously involved multiple researchers with different backgrounds to enrich interpretations of the data through collaborative reflexivity. Interim discussion were held to facilitate a deeper understanding rather than seeking consensus.

## Results

In total, 54 patients were contacted, of whom 16 agreed to participate. Reasons for non-participation included inability to establish contact (after two attempts), feeling unwell, or lack of interest. Interviews lasted between 32 and 53 min, and all were included in the analysis, yielding sufficient rich data to develop meaningful themes.

Participant characteristics are summarized in Table 1. All individuals had undergone abdominal surgery, though the specific procedures varied.

**Table 1:** Participant characteristics

		Number of participants *n* (%)
Sex	Male	10 (62.50)
	Female	6 (37.50)
Age	31–40	2 (12.50)
	41–50	0 (0)
	51–60	3 (18.75)
	61–70	2 (12.50)
	71–80	9 (56.25)
Tumor/organ	Liver	3 (18.75)
	Pancreas	3 (18.75)
	Colon	5 (31.25)
	Rectum	3 (18.75)
	Oropharynx	2 (12.50)

### Themes

Five themes were generated from the analysis. These describe patients’ experiences with lifestyle behavior change in the context of multimodal prehabilitation. Themes included the following: (1) multimodal prehabilitation as a catalyst for lifestyle change, (2) challenges in maintaining a healthy lifestyle post-surgery, (3) the role of motivation and support in sustaining lifestyle change, (4) meaning of health and well-being, (5) unequal access and personal context influence lifestyle choices. Below, the participant’s narrative are presented through verbatim quotes.

#### Theme 1: Multimodal prehabilitation as a catalyst for lifestyle change

Prehabilitation was seen as physically demanding, but manageable. One participant recalled, “I was completely exhausted when I came out of there” (p6), while another emphasized the importance of setting personal limits: “You have to do it, but make sure to set your own boundaries” (p5). Despite the intensity of the program, the experience was linked to a regained sense of agency over their health. As one noted, “It’s something you can control yourself, because you can’t control the cancer” (p10). Others described physical improvements that enhanced their confidence and self-image: “Well, I think it helped me improve my baseline fitness, condition, and strength” (p13).

Multimodal prehabilitation, with its integration of various lifestyle-focused components, provides patients with a new way to engaging with their health. For patients who previously did not engage in regular physical activity, this program played a key role in motivating patients to adopt a more active lifestyle. Participants described the program as empowering: “It felt like a victory” (p13). Improvements in physical capacity were noted and these gains appeared to support ongoing engagement with physical activity. As one stated: “I’m really convinced that being fit before the surgery helps you recover faster” (p10). Positive experiences were frequently shared, and several patients mentioned they would recommend the program to others facing similar procedures.

#### Theme 2: Challenges in maintaining a healthy lifestyle post-surgery

A recurring theme was the discrepancy between patients’ initial motivation and their sustained behavior. While the intention to remain active was high, translating this into long-term practice was often impeded by physical, psychological, and practical challenges. A patient’s ambition to remain active often collided with the restrictive nature of postoperative guidelines, which often advised rest and discouraged early physical exertion. One participant recalled, “Well, they told me, don’t do anything for a while. Take it easy and rest” (p15), illustrating how medical advice could inadvertently stall momentum built during multimodal prehabilitation.

Post-surgical fatigue also emerged as a critical factor. Participants described tiredness not only as a physical consequence of surgery but as a barrier to re-engagement with active routines. As one stated, “I don’t want to be tired like that anymore” (p6). Participants further highlighted the time and energy required to maintain physical activity, which was often difficult to prioritize during recovery. “The downside is that it takes time” (p10), one participant remarked. In addition, the perceived lack of meaningful change also played a demotivating role. “I can’t really say it’s much different than before” (p5), noted one participant.

The discontinuity of support post-surgery was a recurrent concern. Participants described a sense of being left on their own after hospital discharge. One shared, “You get really good support leading up to it, but afterwards it’s like they’re holding you by the collar over a cliff, then that’s it” (p3). Another added, “It ended so abruptly, didn’t it? You go in with such enthusiasm. Then after the surgery, I felt like… yeah, now you’re on your own” (p5). This perceived lack of structured follow-up from physiotherapists or dietitians contributed to feelings of abandonment and uncertainty about how to maintain progress.

Existential reflections emerged, indicating that illness alters the importance of long-term lifestyle goals.. “I just think: make the most of it. Life can be short” (p16), said one participant, hinting at a shift in priorities away from sustained behavioral discipline towards living in the moment.

#### Theme 3: The role of motivation and support in sustainable lifestyle change

Individuals described an intrinsic drive to improve or maintain health, with structured support from healthcare professionals during the F4S program playing a crucial role in translating intention into action. The role of the physiotherapist was cited as pivotal, serving both as a source of practical guidance and as a motivating force. One participant explained, “I received support on all levels, and just that little push to go the extra mile and pay attention to things where I might otherwise overstep my limits” (p14). This external accountability helped participants stay focused and engaged, especially when internal motivation waned.

At the same time, participants recognized the enduring importance of intrinsic motivation in maintaining long-term lifestyle behaviors. As one person noted, “F4S definitely gave me a boost, but that internal motivation, it never really left. Now I can do something with it again, when I couldn’t before” (p13). Another highlighted personal responsibility as foundational: “Of course, you have to be responsible yourself. That’s the first step” (p6). These reflections suggest that while structured programs like F4S can catalyze change, internal drive remains a key determinant of sustainability.

External triggers such as the structured nature of F4S were seen as essential to initiating behavior change. Participants described how simply being enrolled in the program gave them direction and a reason to commit. “If it’s offered to you, then you go for it. You make appointments, you show up, so then you’re involved, you’re in that rhythm” (p15). Another participant added, “It doesn’t need to take months, but just getting a little help to get started again once you’re physically able, that really helps” (p5).

The support received during F4S was also described as confidence-boosting, contributing to a deeper understanding of one’s own physical and mental capabilities. One participant reflected, “I think you can really mean a lot to people, helping them realize their own strength. That’s the beauty of F4S. Not knowing beforehand what you’re capable of, and then achieving more than you thought” (p6).

Social feedback played a motivating role for some. Visible improvements were recognized by others, reinforcing the value of participation. As one individual shared, “After that second surgery and following the F4S program, people said I looked much better than the first time” (p12).

Finally, individual differences in coping strategies influenced how participants engaged with both the challenges and the support systems around them. One participant described a more passive, accepting approach: “I think that’s just in my nature. I always think, we’ll see how it goes” (p16), suggesting that personal outlook may shape the extent to which motivation and support translate into action.

#### Theme 4: Meaning of health and well-being

The experience of illness and surgery acted as a wake-up call, prompting reflection on lifestyle and health behaviors. This awareness often triggered shifts in daily habits, particularly regarding diet and alcohol consumption. One participant described: “Since the oesophageal surgery, the drinking also stopped” (p3); and “They didn’t just take out my stomach, they also changed something in my head” (p3), capturing how physical treatment could spark cognitive and emotional reassessment. Participants emphasized that illness had increased the urgency of maintaining good health: “It was a wake-up call that made it even more important for me to live a healthy life than maybe for other people” (p11).

The value of healthy behaviors was recognized alongside skepticism about their preventive impact: “No matter how healthy you live, it doesn’t give you a certificate of immunity” (p14). Others stressed the importance of doing what one can: “You can’t control everything, but you can influence your body through diet, lifestyle, and exercise” (p13). Small adaptations were experiences as meaningful, as one participant noted: “When I drink less, I sleep better” (p11), indicating how small adaptations were experienced as meaningful.

Engagement in physical activity was frequently associated with improvements in mental well-being. Several participants found that being active created a positive feedback loop between body and mind. “It’s important to feel good physically and mentally. That way, you can age healthily too” (p11). Others highlighted the psychological benefits of sport and movement, particularly in coping with the stress of surgery: “It was really helpful, especially because it was such a nerve-wracking procedure. Exercising gave me a moment to relax” (p13). Even intense exertion was described as rewarding: “That feeling of reaching your goal, even if you’re completely exhausted, that was fantastic” (p6).

Participants’ individual perceptions of what constitutes a healthy lifestyle played a significant role in shaping their health behavior. For example, physical activity was sometimes defined by occupational effort or mobility rather than structured exercise: “I always carry heavy bags. I always walk, never take the car. So, I do strength training” (p16), and “If you work hard, say someone who works outdoors all the time, they can’t do without meat. They just wouldn’t manage” (p9). Similarly, food choices were shaped by notions of “normal” eating: “Just with vegetables and some meat. Meat or fish every day, that’s what I need” (p15). While some changes followed illness and professional guidance, like switching to full-fat products based on how they made one feel, many decisions reflected a blend of personal logic, tradition, and experience.

#### Theme 5: Unequal access and personal context influence lifestyle choices

Participants’ ability to adopt and sustain lifestyle changes appeared to be shaped by their financial situation. Although participation in the multimodal prehabilitation program itself was free of charge, participants noted that follow-up care was financially out of reach. One participant shared, “I thought about it and even discussed it with the physiotherapist, maybe I could follow a program after surgery at my own expense. But that’s just way too expensive” (p3). In contrast, the fact that multimodal prehabilitation came at no cost made it easier for participants to engage: “Then money comes into play. As long as you don’t have to pay for it yourself, that makes a difference, right?” (p9).

Beyond finances, participants’ lifestyle decisions were also influenced by their broader life context and how they prioritized health. Managing illness was tightly connected to future hopes and personal meaning. One participant reflected, “I dream that at some point the ‘palliative’ label could be taken off my illness, that it could be called something else, so that I can start seeing life in a different light again” (p14).

The social environment also played a crucial role. Participants emphasized that support from others helped them stay motivated and emotionally resilient. “You just need people. They’re important too. That’s also part of well-being” (p6), said one, while another added, “It’s indispensable. If you have to do this alone, it’s just not doable” (p13). Family and friends were sometimes described as a key source of support: “I get emotional when I think of how my friends supported me. I have three brothers, but there are two I’m especially close with. If you only knew everything they did for me in the meantime” (p4).

Shared activity was also highlighted as a motivator. “That really works better, I think. You encourage each other without realizing it,” one participant said, describing how exercising with others helped sustain effort (p7). Still, maintaining new habits was not always easy. One participant noted, “Sometimes I still think, I’m going to eat fruit every day: an apple, two pieces of fruit. That works for two days… and then not anymore” (p16).

## Discussion

The findings from this study highlight the role that multimodal prehabilitation plays in shaping patients’ health behaviors before and after surgery. A central theme was the perception of the program as a dual-purpose intervention. It was consistently described not only as physical preparation for surgery but also as a stimulus for broader lifestyle changes. For many, the structured support provided a crucial sense of agency and psychological benefit during a vulnerable period, leading to rediscovered enjoyment in physical activity and enhanced self-efficacy. However, sustaining these behavioral shifts postoperatively was challenging. Factors such as fatigue, restrictive medical guidance, and the abrupt withdrawal of support undermined long-term maintenance. Therefore, providing structured follow-up after surgery, with the possibility of transitioning to primary care, may enhance continuity of care and offer psychological and practical support during physical recovery and behavioral adjustment [11].

Physical activity following major surgery often declines substantially, with many patients remaining below baseline activity levels for months or even years [12]. A 12-month prospective study with patients who received low anterior resection for rectal cancer found that total physical activity remained significantly below preoperative levels even at 1 year post-surgery [13]. Similarly, a cohort study for colorectal cancer surgery patients revealed that more than half had not regained pre-operative physical functioning at 6 months [14]. This prolonged inactivity poses a significant public health concern, given the well-established links between insufficient physical activity and the risk of chronic disease and poor health outcomes. These findings underscore the necessity of behavioral prehabilitation programs, and focus on regaining and sustaining physical activity after surgery. A smooth transition from clinical care to daily life is essential to support this process. This aligns with our findings, where patients highlighted the lack of guidance on post-surgical activity.

While the experience of illness heightened participants’ awareness of the link between lifestyle and well-being, their ability to act on this awareness was often constrained, leaving some less active patients ‘stuck’ in the awareness phase, consistent with the I-Change model of behavior change [15, 16]. These findings align with recent meta-analyses indicating that while prehabilitation may produce clinically meaningful improvements in the preoperative and immediate postoperative phases, its effects tend not to persist long-term when compared to usual care [17]. This aligns with Verplanken and Orbell’s theory [18], which emphasizes that while awareness, attitudes, and intentions are important, health behaviors are largely governed by automatic habits triggered by contextual cues embedded in daily life. Consequently, future interventions may benefit from integrating behavior-change techniques such as goal setting, regular follow-up to enhance accountability, and approaches aimed at sustaining motivation over time.

Participants’ lifestyle behaviors were also influenced by personal, social and systemic factors. Financial constraints, limited social support, and varying access to resources created unequal opportunities for sustaining health-promoting behaviors beyond the prehabilitation program. Accounting for system-level barriers such as insurance limitations, low health literacy, or lack of paid sick leave is essential for ensuring access to follow-up care. While multimodal prehabilitation is offered regardless of health insurance or additional personal costs, our study suggests that follow-up care remains less accessible. Therefore, it is crucial to account for health inequity and inequality when developing public health interventions that are tailored to be implemented within one’s own environment.

External support emerged as critical. Participants emphasized that structured encouragement during multimodal prehabilitation supported healthy behaviors, echoing prior studies linking supervised preoperative exercise programs to higher physical activity post-surgery [19, 20]. Maintaining these changes without ongoing professional support proved challenging [20]. Moreover, while illness can act as a “teachable moment,” the post-cancer period may be better framed as an opportunity for ongoing dialogue about priorities and needs rather than assuming automatic behavior change [21], underscoring the value of incorporating prehabilitation principles into survivorship pathways.

Metabolic research further underscores the importance of lifestyle changes for cancer prevention and management [22], yet the journey towards sustainable behavior change remains complex. The average time needed to establish new habits, around 66 days [23], combined with the fact that 50% of patients exhibit reduced physical capacity even three months postoperatively [24], suggests that behavior change timelines need to be more realistic and tailored. Our findings align with these insights, demonstrating that physical recovery trajectories, motivational dynamics, and external structural factors all significantly influence the maintenance of health behaviors after surgery.

This study has several strengths and limitations that merit consideration. A key limitation is the recruitment from a single academic medical center. While this restricts the statistical generalizability of findings, our primary aim was to obtain rich insights aligned with RTA. We argue that while the organizational context is local, the identified behavioral mechanisms possess transferability to other surgical oncology settings. Steps were taken to enhance the diversity of the sample by including patients across different cancer types and care pathways.

Regarding the timeframe, patients were interviewed at least 3 months post-surgery. While this introduces the potential for recall bias, this timing was deliberately chosen to capture the full recovery trajectory and the sustainability of lifestyle changes, rather than focusing solely on the acute postoperative phase. Additionally, social desirability bias may have occurred, as participants were interviewed by members of the prehabilitation team; this was mitigated by ensuring the interviewer (LA) had no prior treatment relationship with participants.

Methodologically, rigor was strengthened by member checking at the transcript level to ensure data accuracy. We deliberately did not seek participant validation for the final *themes*, as RTA views themes as conceptual syntheses generated by the researcher rather than direct participant accounts. Finally, we acknowledge that the interview guide, developed by physiotherapists, may have emphasized physical activity over other lifestyle domains such as nutrition or sleep. Indeed, physical activity emerged as the most frequently discussed topic, which may reflect both the interview prompts and its prominence within the multimodal prehabilitation program.

## Conclusion

These findings highlight the need for more sustained interventions focusing on motivation and long-term health behavior to support behavior change following major cancer surgery. Multimodal prehabilitation programs appear to offer a critical starting point, but without continued professional guidance and realistic timelines, long-term adoption of healthy behaviors remains difficult, particularly for less active patients. To ensure that the short-term benefits of prehabilitation lead to sustainable health behavior, future efforts should focus on fostering long-term behavior change by leveraging personal motivation, engaging external support, strengthening habit formation, and addressing limiting factors such as fatigue, loss of support, and insufficient medical guidance.

## Supplementary Information

Below is the link to the electronic supplementary material.

ESM 1 (DOCX 21.0 KB)

## Data Availability

No further data or materials are available. The raw data are in the form of audio recordings and transcripts and there are ethical restrictions on sharing them.
