# Supplementary material for: Lifestyle perceptions following multimodal prehabilitation in patients undergoing cancer surgery: a qualitative study
Source: Support Care Cancer. 2026 Apr 21;34(5):450. doi: 10.1007/s00520-026-10661-7 (PMC13095926; doi:10.1007/s00520-026-10661-7)
Supplement: Supplementary file 1 — (DOCX 21.0 KB) [file 520_2026_10661_MOESM1_ESM.docx]

**Supplementary File I: Interview guide**

Topics:

1. **Knowledge**

To provide insight into experiences, ideas and opinions about physical activity in relation to diagnosis/surgery.

1. **Attitude and Motivation**

To provide insight into the reasons for being physically active and what the advantages and disadvantages of this may be.

1. **Self-Efficacy**

To provide insight into management of lifestyle, environmental factors and the influence of the social environment.

1. **Future Perspective**

To gain insight into the sustainability of behavioral change and setting goals for the future.

| **Interview guide** | |
| --- | --- |
| Starting Question | **Can you share what aspects of the Fit4Surgery program you found most memorable?** |
| Healthy Lifestyle in Relation to Health | **Objective:** To gain insight into the patient's beliefs about lifestyle-related health behaviors and the values attributed to them. |
|  | **Can you tell us what you understand by a healthy lifestyle?** |
|  | 1. To what extent do you think health can be influenced by a healthy lifestyle? 2. Can you give examples of healthy and unhealthy behaviors? 3. What are the advantages and disadvantages of a healthy lifestyle? Can you provide examples? 4. How much value do you place on a healthy lifestyle? 5. Which lifestyle factors do you believe contribute to a healthy lifestyle? |
| The Meaning of Physical Activity | **Objective:** To gain insight into the patient’s perceived meaning of physical activity. |
|  | **What does physical activity mean to you?** |
|  | 1. What do you see as possible advantages or disadvantages of being physically active? 2. How do you feel when you are (or have been) physically active? 3. Has the Fit4Surgery program made you think differently about (the importance of) physical activity? 4. Do you see a connection between physical activity and health? Can you explain it? Do you have any examples? |
| Physical Activity and Fit4surgery | **Objective:** To gain insight into how Fit4Surgery has contributed to behavior change and which elements the program might still be missing that could be beneficial. |
|  | **Has the recent period caused a change in the amount of physical activity you do?** |
|  | 1. If yes, what were the main reasons for this? If no, why not? 2. Can you name what you have learned from the past period? 3. What have you specifically learned from the Fit4Surgery program? 4. What would you advise others regarding physical activity who are about to go through the same program as you? |
| Physical Activity and Motivation | **Objective:** To gain insight into motivation (perceived facilitators and barriers) for engaging in physical activity. |
|  | **What motivates you to be physically active?** |
|  | 1. Has your motivation changed since your surgery? Why or why not? 2. Has your activity level changed after the surgery? Can you explain why and in what way? 3. What do you need in order to stay motivated to be physically active? 4. What do you experience as barriers or facilitators to being physically active? |
| Carrying Out Physical Activity | **Objective:** To gain insight into integrating physical activity into daily life. |
|  | **How physically active are you currently?** |
|  | 1. Are you physically active? What do you consider physical activity? Can you give examples? 2. At what times do you engage in physical activity? 3. Is performing physical activities part of a fixed weekly routine? 4. Do you receive enough support from others in this? |
| Goals and Physical Activity | **Objective:** To gain insight into how patients set short- and long-term goals related to physical activity and their perceived importance of these goals. |
|  | **What goals do you have regarding physical activity in the future?** |
|  | 1. How do you plan to achieve them? 2. Why do you have (or not have) these goals? 3. What can physical activity bring you? 4. What are potential pitfalls, and how do you plan to handle them? |
| Closing | **Do you have any additional comments?** |
